# Medico-Legal Studies on the Perversion of the Moral and Affective Faculties in the Precursory Period of General Paralysis

**Published:** 1861-01

**Authors:** A. Brierre de Boismont


### Art. VI.—MEDICO-LEGAL STUDIES,

### ON THE

### PERVERSION OF THE MORAL AND AFFECTIVE FACULTIES IN THE PRECURSORY PERIOD OF GENERAL PARALYSIS.*

By A. Brierre de Boismont.

(*Read before the Institute of France* [*Academy of Sciences*] *24th September*, 1860.)

There are few phenomena of greater interest to the physician and the moralist, than the change which insanity impresses upon the character, the disposition, and the temperament. It might be said that a magic wand had brought about the metamorphosis, so often does this happen rapidly. That man who was grave, punctual, reserved, economical, and devoted to others, becomes

* Translated from the French:—*Annales d'Hygiène Publique et de Médecine Legale*, Octobre, 1860.

giddy-brained, vacillating, dishonest, wasteful, and an egotist. This man, whose discretion had gained the confidence of all, whose winning manners and happy disposition had been the charm of domestic life, casts to the wind the trust which has been reposed in him, and becomes malignant, brutal, a railer and insolent. Can it be that a little more blood flowing towards the brain, or a more lively excitation of the nervous system, gives rise to these anomalies, and throws up to the surface evil instincts aforetime hidden, as it would seem, from observation? But are not these elements of causation the same that we invoke to explain acts of heroism, sublime deeds, and creations of genius? Evidently if the elements are similar, they must be modified or combined differently in the two cases. In the arrangement of these modifications rests the enigma of the cause of those pathological lesions of which, up to that point, the effects have been demonstrated; and this enigma is not of a nature to be quickly solved. As to the anatomical lesion that is found so constantly in general paralysis, we cannot attribute to it the moral disorders witnessed in the progress of the disease, since these are observed in other forms of mental alienation which, up to the time when these disorders have become apparent, have not presented any appreciable anatomical change; or if such a change exists, it has no relation to the moral symptoms referred to. Moreover, in paralytic insanity the moral disturbance occurs many years before the malady is suspected.

Insanity manifests every moment the transformations spoken of. They are common in the paroxysms of maniacal excitement which resemble drunkenness; they are observed also in the moral insanity of the English, and in our reasoning manias (*folies raisonnantes*), &c.

We shall study these transformations here solely in relation to paralytic insanity, better known under the name of general paralysis of the insane.

One of the first and most constant of the appreciable indications of intellectual disturbance is the change of character, which consists ordinarily in greater irritability, and in movements characteristic of impatience, anger and violence. If the disposition be naturally of this quality, the characteristics become much more conspicuous, exciting the attention of relatives and friends. This moral perturbation may persist six months, or one, two, or three years, without any concomitant symptoms.

Under other circumstances, the derangement of the mind is announced first by extravagant talk. A clerk, necessarily in contact daily with hundreds of persons whose observation he could not escape, was invited to a wedding. All at once he began to converse in the most extraordinary manner, and exhibited a mobility of ideas that could not be checked. Sometimes the disorder of the faculties is revealed by acts. A wife, an excellent manager, made purchases out of all proportion to her resources. She despaired, and attempted to kill herself. Shortly afterwards general paralysis became evident. Often we have noted, as the first indication of evil, the menace of suicide.

The change of character may present very varied shades. Four years before the invasion of paralysis, a man who had up to that time been habitually firm, became irresolute, uncertain, and wept readily, although he still managed to conduct his business well for a period of three years. At the end of that time another change came over his disposition ; he became irritable, fiery, and choleric. Paralytic insanity succeeded these two metamorphoses.

It is not uncommon to observe, in place of the choleric irritability just mentioned, a placidity or an apathy which results in the individual's being diverted from all serious occupation. We have observed six instances of this kind. The relations, amazed, ceased not to comment and to animadvert upon the grave consequences of this conduct ; the patients calmly admitted the soundness of the reasoning, but averred that it was impossible for them to do anything.

These transformations of character lead us to speak of the important facts to which we called attention, thirteen years ago, in the *Gazette Médicale.** Long before the paralysis appears (we have noted facts extending back from six to seven years), perversions of the moral and affective faculties become manifest among certain individuals, which do not prevent them from fulfilling the duties of social life, and of acquitting themselves of their callings. The families, surprised and distressed, whisper among themselves of acts of indelicacy, improbity, and debauchery for which no antecedent had prepared them. They extenuated the wrongs, they paid the penalties, they stifled their complaints, until this long and secret martyrdom terminated by the breaking out of the disease.

"Obs. I.—A superior official of a great establishment had performed his duties with capacity and zeal almost up to the moment when he was placed under my care. Nevertheless, the details given me by his wife left no doubt that the perversion of his faculties was of long standing. Previously generous and of chaste habits, he had for more than six years manifested sordid avarice and unbridled licentiousness. His wife had ceased to demand from him money for household purposes, because when asked he would become so violent as to cause her to fear mischief. With the progress of his malady, his avarice prompted him to most humiliating acts. He would not pay money due, assert-

* *Quelques Remarques sur la Paralysie générale des Aliénés.* (*Gazette Médicale,* du 22 mai, 1847, p. 381; *Revue Médicale,* 1846.)

ing that he had already paid it; and in the end he had stolen objects from the houses of his acquaintances. Even these last acts were looked upon as eccentricities, no one suspecting the disorder of his mind; and it was not until the life of his wife had been placed in peril by ill-usage that it was resolved to place him in an asylum. There he remained more than five years; his motility at the time of entrance presenting but slight signs of disturbance, but the memory being manifestly enfeebled.

"OBS. II.—Some time after, I was consulted about an individual whose pilferings at a public auction some years before had excited great attention. The observations which I had collected at this time had caused me to think even then that the man was under the influence of the precursory period of general paralysis. I avow that this interview roused my curiosity to the highest degree. I was almost certain that I should find a paralytic lunatic. No information had been given to me. The first words the patient uttered when I entered his room revealed the nature of the affection from which he suffered, and its long standing. The pronunciation was in fact embarrassed, there was manifest incoherence, the physiognomy was, as it were, petrified, and the gait was sluggish. More than eight years had passed since his pilferings had been first noted, and they had never entirely ceased, but it was only a few months before that mental alienation had been recognised. As this patient had lucid intervals and there was no fear of wounding his feelings, (since in cases of this kind the weakening of the intellectual mechanism destroys the susceptibility and renders the patient indifferent to anything), I turned the conversation upon the acts which had led to his arrest. He spoke tranquilly about them and said, 'The men who examined me and placed me in a prison were imbeciles who knew nothing of our profession. It is an usage among us when making an inventory, and this usage is called the *quota G* [*la cote G*] to choose an article, generally of little value, and here are two that I have thus obtained,' he concluded, producing a beautiful meerschaum pipe and a tobacco-pouch embroidered with gold. He then became again incoherent. There could be no doubt respecting the nature of the disease, and I withdrew. A few months afterwards I learned that the general paralysis terminated fatally."

Since the insertion of this note in the *Gazette Médicale* my collection of similar facts has been augmented, and as this subject is not less interesting for the history of the disease as well as for legal medicine, I propose to report several examples taken from one hundred observations collected by myself, and of which the results were communicated to the Medico-Psychological Society.*

"OBS. III.—*Symptoms of general paralysis of fifteen months' duration; malversation of funds seven months after the first indications*

* *Recherches cliniques sur la Paralysie générale* (*Annales Medico-Psychologique,* 1859, p. 294.)

*of disease.—Commencement of legal proceedings.—Progress of the disease.—Death.*

"A railway clerk was confided to my care in 1847, to be treated for general paralysis, which had reached an advanced stage. The stammering was marked, there was inequality of the pupils, and feebleness of the inferior extremities, and the gait was vacillating. The memory was weakened, yet the patient conversed rationally, but if he were interrogated upon his health, his position, or his profession, great exaggeration was noted. According to his own account he was perfectly well, gained much money, and performed fully the duties of his situation. After the fashion of many of these patients, he gave no attention to external events; he did not manifest any astonishment that he was placed in an asylum; he ate with avidity, and took no part in that which passed around him. On examining his accounts an abuse of confidence was discovered, and legal proceedings were commenced against him. He was requested in my presence to give an explanation of the malversations he had been guilty of, and to state the use to which he had applied the missing funds; but the only reply which he would give was this: 'That money belonged to me, I earned it by my assiduity in work, and by the improvements which I have introduced into the establishment.' It was in vain that attempts were made to convince him of the falsity of this reasoning; he would repeat imperturbably that the money was his. This opinion will not surprise alienists, because they know that many of these unfortunates have the conviction that they are millionaires, or that everything belongs to them. It was important to ascertain when the first indications of this patient's malady had become manifest. After some trouble I ascertained that fifteen months before, a change in his habits had been observed. Little by little it had been noted that the memory was occasionally defective, that he entertained exaggerated ideas of his position, and that he was affected by a momentary embarrassment in his speech; but as he fulfilled the duties of his place with regularity, these symptoms had not been much heeded. The subtractions had gone on for eight months, but the inquiry concerning them was of necessity abandoned on account of the rapid progress of the general paralysis. The incoherence became complete, he barely responded when addressed, he kept himself erect with difficulty, and he succumbed to the cerebral marasmus after two months' residence in the asylum.

"Obs. IV.—*Licentious acts and unfortunate speculations during a period of about six months.—Intellectual symptoms announcing general paralysis, but no lesion of motility.—Mandate of arrest.—Rapid progress of the disease.—Death three months afterwards.*

"A merchant, aged forty-six years, whose conduct had always been honourable, was brought to my establishment in 1846, on account of acts of licentiousness of which he had been guilty over a period of half a year, and which were so entirely opposed to his usual habits that his family, painfully affected by his conduct, thought that it must be attributed to some mental derangement.

"For several months, moreover, he had given himself up to speculations, of which many had failed. Even at the time when attention had been aroused by his disordered actions, nothing in his discourse and manner of living had excited any suspicion of mental disturbance. He visited the Bourse daily, had numerous communications with persons of his calling, but none of them had perceived his mental state, or, at least, no one had pointed it out.

"When he was brought to me, he neither showed any emotion, nor manifested any astonishment at being transferred to an unknown house. I spoke to him first upon the acts which had led to his being placed under control. He answered, speaking carelessly, and as if the matter did not concern him, 'That alarm had been too readily taken, and that everything would be explained.' I interrogated him afterwards about his business, and the position of his affairs. To these questions, which did not seem to surprise him, he to all appearance responded rationally but somewhat evasively, and gave no explanation. I referred more particularly to certain of the points on which I sought information, and he said, 'My business affairs, like other commercial matters, are both good and bad, I have not to complain of them. My family behaves well to me; my position is satisfactory, and my health is very good.' I attempted to question him more closely, but he then responded, 'I do not know; I cannot call to mind.' Not being able to elicit anything more from him, I terminated the conversation, and he wished me to allow him to visit the Bourse. This request not being acceded to, he left me, as if the refusal were a matter of trifling importance, and went into the garden.

"During this conversation it was evident to me that the attention was enfeebled, the memory confused, and consciousness modified, but I did not observe either embarrassment of speech, disorder in the movements, or manifest incoherence. I concluded, however, that the man was under the influence of general paralysis, and I stated to his relatives that grave consequences were to be apprehended, not only to his life, but also to his fortune.

"The examination of his books was a thunder-stroke. They were badly kept, showed great omissions, and the only certain information to be obtained from them was that ruin was imminent. The commercial position of the unfortunate man presently, however, assumed a more serious cast. The judges of the Tribunal of Commerce pronounced on his affairs a verdict of fraudulent bankruptcy, and directed his arrest; and an officer of the court presented himself at my establishment with the necessary mandate. I conducted him to the patient, in whom, in the space of three weeks, the following changes had taken place. His memory was entirely lost, and he could not respond to any questions put to him. His look was stupid and his figure immobile. Already embarrassment of the speech might be noted, and feebleness of the legs showed positively that he suffered from general paralysis, and that the habitual excitation of his life had been masked by mechanical movement. I declared to the officer that in the state in which the patient then was, I could not permit him to execute the mandate; and I added that from the rapidity with which the affection had proceeded, it was almost certain that a serious termination would very shortly occur. I

prepared a certificate to this effect, and forwarded it to the President of the Tribunal of Commerce, and the arrest was adjourned until the re-establishment of the patient's health. Three months afterwards this patient died in the last degree of brutishness and marasmus."

Disorders of the intelligence, and perversions of the moral and affective faculties may exist long before the appearance of lesions of motility, and even pass unperceived for months or years, because the patient speaks little and conceals his acts.

Obs. V.—*Change of character during a period of two years.—Irregular conduct extending solely over a few months.—Recent extravagant acts.—Bankruptcy.—Judicial inquiry.—Singular answers—Belief that the individual simulated insanity.—Examinations to ascertain his mental state.—Report proving the disordered state of the mental faculties without lesion of motility.—Sudden occurrence of embarrassed speech and of myriads of transitory ideas.—Paralytic dementia, and rapid progress of the affection.—Death in three months.*

M. Henri, a merchant, aged fifty years, and of good constitution, was brought to my asylum in 1852 for mental disorder, which was said to be of recent date. At the time of his entry the physiognomy announced health, but the gaze was without either fixity or attention. The walk was firm, the attitude erect, and the limbs manifested nothing abnormal. When he was questioned, it was impossible to obtain from him any connected reply: he heard without appearing to comprehend. Seeing an open door, he immediately endeavoured to escape through it, but, on the door being closed, he did not make any observation or complaint, and he expressed no desire to return home.

I thought that the patient was suffering from dementia with general paralysis, but it was impossible to demonstrate the changes constituting the latter affection. The gravity of the case led me to doubt that the mental disturbance was so recent as the relatives believed. I questioned them, and ascertained that an alteration in the patient's character had been observed for two years. He had become incommunicative, answered abruptly the questions put to him, and would not give any explanation of this change of disposition. Very regular in his habits and of exemplary life, he had begun, many months before, to absent himself from home without its being known where he went, and it was not until he had been carefully watched that it was discovered he frequented places of evil resort. His taciturnity was attributed to the state of his business, which was bad. These changes, in fact, dated back to a time when his brother had become bankrupt, and when he himself had in consequence been obliged to close his accounts; but it was only when he had been guilty of sundry extravagances that disease was recognised.

The heavy losses occasioned by these two failures, and the absence of entries in the books, gave rise to most unfavourable surmises, which became still more serious on account of the patient's conduct. When he was requested to give the information required to unravel his affairs, he would respond in a jovial tone—"*Very likely: I want nothing, I do nothing now, and I do not wish to do anything; I will see to it pre-*

*sently. I do not require anything; all my affairs are in a prosperous state.*" More commonly he said, "*I have done that which is customary in commerce; all will be explained, all justified.*" These brief answers were made without any embarrassment of speech, hesitation, or stammering, but in a mocking manner, and it was thought that he simulated insanity.

This opinion, which did not lack weight, induced me to examine the patient daily with great care. He did not speak to the other boarders, but often played at piquet. After a sojourn of many months, it was noticed that he counted badly, and that he became forgetful, the gait, the limbs, and the tongue still maintaining their normal state. Sometimes it appeared to me that he hesitated in the articulation of certain words, but several months passed without my being able to satisfy myself upon this point. He walked every day many hours with a firm step, then he would sit down in a corner of the hall, reading or seeming to read one book, the leaves of which he rarely turned. When interrogated about what he read, he would answer, "*I know what I read,*" and would not add another word. Twice he had an involuntary evacuation.

As the idea of simulation was still entertained, I was directed to make a report on the case. Founding an opinion upon the enfeeblement of memory, the errors of calculation at piquet, the abandonment of an amusement which had appeared to gratify the patient, the failure of attention, the indifference with which he received his family, his placid air, his singular smile, his promenade in a particular corner of the hall, his incessant reading, the two involuntary evacuations he had had, and the diminution of motility and cutaneous sensibility, I declared that he was attacked with dementia, that general paralysis, which I had expected on his entry into the asylum and in the existence of which I still believed, was not yet manifested by any characteristic signs, but that it was impossible to believe in simulation. The *statu quo* was maintained eighteen months; my opinion was acted upon, but as the physicians who frequently examined the patient on the part of the authorities could not distinguish any embarrassment of speech, trembling in the limbs, or incertitude of gait, and as he appeared to laugh at the questions put to him, confining his answers to the words we have already recorded, and turning his back upon the interrogaters when the questioning was prolonged, the opinion of simulation, although shaken, still existed. Some admitted there had been derangement of the mental faculties, but all thought that he knew what he was about.

On the 28th March, 1853, when I made my usual visit, he came to me with a precipitous step, and somewhat uncertain gait, saying that he was very well. I was struck with a very marked embarrassment in his speech, he stuttered at every word. His aspect was stupid, he saluted me at different times after the fashion of a clown in a pantomime, and afterwards drawing me aside, *requested me, in a low voice, to lend him from three to four millions.* Then he returned to his corner and recommenced reading the book he never quitted, and which he most commonly held in the hand with the pages reversed. For five days the stammering was very marked. He *spoke once more of the*

*three to four millions*, and then his taciturnity and ordinary immobility returned. From this time the disease made rapid progress. M. Henri became more feeble upon his legs, soiled himself, wasted, and in three months succumbed to marasmus."

The case that we shall next record has been observed from the first symptom of disorder even to the fatal termination. It has been possible, consequently, to demonstrate with exactness a primary derangement characterized by exaggerated fears regarding the health, with return to reason for three years. Then a second disorder having the characters of general paralysis, first under the form of ambitious mania, and subsequently under that of paralytic dementia.

Obs. VI.—*Exaltation of an hypochondriacal character.—Cure, but with an exaggeration of the habitual disposition remaining.—Appearance of general paralysis at the end of three years.—Death.*

M. T——, aged fifty-two years, and of good constitution, had always been irascible, hyperbolical, irritated by the slightest remarks, and could not bear the most trifling discomfort. This disposition is very generally that of his compatriots. For many years he was subject to an eczematous eruption situated on the inner surface of the thighs. He had been advised to use sulphurous lotions. The application cured the cutaneous disease, but he asserted that in consequence of this treatment an intercostal neuralgic affection arose which radiated to the neck and vertebral column. Four months of medication gave him no relief, and he entered a great establishment in Paris. The violence of his lamentations, his paroxysms of anger, and his continual menaces of death, induced the physician who had care of him to recommend him to go into a lunatic asylum. When he came to me he complained of the most intolerable pains. According to his own account, he was tortured, wasting away, lost, and it remained for him but to die. If he were touched, he shrieked as if he were being flayed. After the employment of prolonged baths for a few days he became somewhat better, but as soon as he felt the least return of his disorder, he uttered piercing cries, wept, groaned, rolled upon the ground, and wished to kill himself, but without ever making a real attempt. This patient was the torment of the house. He called for medical aid day and night, and when remonstrated with for his unreasonableness, he answered, "What does your sleep matter? I am dying, and the physician is the property of the patient who is threatened by death."

Little by little this great excitement subsided, and at the end of three months the patient was sufficiently well to take excursions in Paris. During the last six weeks of his stay with me he went frequently to the theatres and into the country with members of my family, and there remained but a more manifest degree of that habit of exaggeration which is peculiar to certain portions of southern countries. M. T—— spoke of his fortune, his acts, his conduct, and his knowledge, with expressions so hyperbolical that we all said, "He is menaced with general paralysis; he will have the mania of millions." He proposed unceasingly operations which would, he asserted, bring him in great sums. A real amelioration had, however, taken place in his state; his neuralgic pains were bearable, he attended to his own interests, and he determined to return home in order to resume his business. When he quitted my care he did not manifest, and he never had manifested, any indication of embarrassment of speech, feebleness of the limbs, diminution of sensibility, or alteration of sight. M. T. was observed most narrowly from the intimate relations which had been established between us.

A year and a half after his departure, he returned to Paris, and called upon me. Apart from his habitual exaggerations, which were a little more marked, he possessed his full intelligence, he executed his business, but in transacting it he displayed an obstinacy which debarred all counsel, and often prevented success. He expended much uselessly; he made purchases heedless of cost, and made expensive gifts. He was not submitted to any examination. For the rest, his conduct manifested nothing of moment, and there were no symptoms of the disorder we feared. One of my sons, some time after, passed a month with him, and he observed solely outbursts of passion and paroxysms of extreme anger on the slightest occasions.

Three years and a half after leaving my establishment, M. T. again came under my care, and for general paralysis. It would seem that the neuralgic pains which had attacked him some years before his first admission, and which had greatly diminished, without ceasing entirely, had now been lost altogether for about a year. This disappearance was followed presently by a total change in M. T.'s character. In place of the obstinacy and anger which habitually characterized him, he had become gentle, but entirely unable to conduct industrial operations. He wished to hear no more of them, and when his children besought him to let them act, and represented to him the losses resulting from his inaction, he replied, "We shall recommence our operations next season. What does it matter that we lose now 50,000 francs, when we shall regain 500,000 in a year?" He saw everywhere prodigious profits, and believed himself to be the possessor of fabulous sums. Presently he committed eccentric acts; he visited the *cafés* dressed in the most singular manner, and set himself up as a good-fellow, speaking to every one. He squandered money as an useless thing. His memory failed, and hesitation and stammering were noticed. The children, who had been forewarned of the nature of the malady, then confided him anew to my charge.

A few days after his arrival, MM. Calmeil and Parchappe saw the case in consultation, and diagnosticated a grave paralytic dementia in an advanced stage, and which would have a rapid course. During the first five or six months he passed with me, he went backwards and forwards, packing his trunks at intervals in order to return home, but abandoning this idea on the first remark. His cutaneous sensibility was very much blunted; he did not move when pinched; he grasped indifferently; but he preserved still his ideas of riches, saying from time to time that he should have a magnificent year, gaining hundreds of thousands of francs—nay, millions. But the activity he had retained was insensibly replaced by apathy; he wavered upon his legs and fell occasionally. At this time he spoke no more of grandeur and riches but as far off; he passed his days upon a chair, sad, and with a melancholy aspect, saying that he was ill. One day, although the memory was nearly gone, he cried out that he was becoming mad. In the last months of his sojourn in the asylum, he never quitted the corner of the room in which he was placed. His eye was fixed, and without expression; he no longer recognised his relations, or else he confounded one with another, and he responded with difficulty to the questions put to him. The speech was much embarrassed, and there was very marked incoherence. He was led as an infant, and it was with difficulty that he held himself erect. The change which he had undergone was so great, that we thought when he returned to his own home he would succumb in a few months. He lived, however, nearly two years, after having undergone spasmodic contractions, rigidity of the limbs, and wounds and scars which healed and broke out again, and were again healed.

This observation, of which the subject has never been lost sight of, is highly interesting from more than one point of view. First, we can follow step by step the long incubation of the general paralysis to which this unfortunate organization was, so to say, fatally predisposed. A primary derangement of mind, characterized by exaggerated hypochondriacism, announced the approach of the evil. This fact confirms M. Baillarger's opinion upon hypochondriacal delirium considered as a precursory symptom of general paralysis.* A stationary period occurs from the efforts of nature, but it is foreseen that sooner or later the paralysis of millions will be developed; the intellectual rudiments exist, those of motility alone fail. Three years pass in the midst of motiveless outbursts of passion and anger; the patient's affairs are conducted solely under pressure, and they are damaged every moment by false judgments, a capricious will, and acts, which without being stamped with madness, are often hurtful. Fortune may be compromised and often lost in consequence of the determinations arising from a brain placed in similar conditions; but it is necessary to look idly on and to contemplate the event without the power of preventing it, as the law does not permit, in this case, any measure of conservation so long as the individual speaks reasonably. Lastly, the moment comes when the evil is matured and breaks forth. Here we note a phenomenon which has often been observed in cases of a like character—to wit, the neuralgia which had endured several years ceases entirely. Almost at the same moment a radical change takes place in the patient's character, and his disposition becomes different in every respect to that which it had previously been. Although most commonly insanity exaggerates the qualities and defects of an individual, there are cases which prove that occasionally it substitutes a new character for the old one. This phenomenon was followed by the symptoms of paralytic mania in its ambitious form; and this, after existing many months, was succeeded by paralytic dementia, mixed temporarily with a little melancholy depression, in which the patient succumbed after a struggle of more than two years.

* *Académie des Sciences,* 17th September, 1860.

The cases we have recorded do not leave any doubt upon the changes which the character, the disposition, and the habits may undergo from paralytic insanity. In the analysis of our one hundred cases of general paralysis, it is found, when the *third category* is studied, comprehending *disorders of the understanding preceding those of motility,* and numbering forty-two observations, that one of the first and most constant appreciable disorders of the intellect is a change of character which ordinarily consists in a greater degree of irritability, impatient movements, anger, and violence. This was observed in three-fourths of the cases.

In a much more restricted number of individuals the disease was, on the contrary, preceded by a calm, placid, indolent and apathetic state. These individuals reasoned well, and admitted that they ought to work, to act, to attend to business, but between speech and action there was an abyss which they could not leap over. One of the earliest examples that we have noted of this change, was that of the chief gardener of a wealthy mansion. For many years he had done his duties, which were solely of a routine character, well. His activity was great, and sufficed for everything. Gradually he became silent and relaxed his superintendence; he complained of confusion in the head; he still reasoned well, but he confessed that rest would be useful to him. He was placed under my care for two months. In the first he improved; in the second he conversed with great lucidity on his horticultural labours. It was interesting to listen to him, and he appeared to be very wishful to return to his occupation; he indicated, moreover, certain improvements to be effected. We thought that he was completely restored, and he was discharged and immediately returned to his duties. Some time after we had news of him. On his return to the country, it would seem, that he discussed what he had to do, but he was unable to execute anything, or to give any orders; he was always undecided; and it became necessary to dismiss him.

Another example was that of an architect, also confided to our care. M. had a young wife who was very much attached to him. Two years after marriage she perceived that her husband became incommunicative, and that he occupied himself no more with his projects; he promised to look after them, and recommence work, and to go about, but kept to the house. To the representations of his wife he responded that he desired nothing more than to work, but he continued to do nothing, and this state persisted a year. It was observed that he became more irritable and capricious, and that at times he was forgetful. At times, also, he stammered. About this period he was brought to me. He was then in the second stage of general paralysis, the symptoms of which had been clearly apparent for a period of from five to six months.

He became more gentle, but he always remained apathetic. After a sojourn of six months, his family finding him better, removed him home. The stationary condition persisted more than a year, then the disease made rapid progress, and the patient succumbed at the end of two years.

In place of choleric irritability or of a seemingly rational apathy, or with one or the other of these states, graver symptoms may be manifested, such as perversions of the moral and affective faculties. Families are distressed with these changes and do not foresee that they arise from a malady which is very often fatal. In fact, the individuals affected with it continue to perform the duties of social life. Acts of indelicacy, of dishonesty, of debauchery, &c., are noted from time to time, and every effort is made to conceal or make reparation for them; and sometimes the scandal is so great that the law steps in and punishes the unrecognised lunatic.

It is in this precursory period of general paralysis, which may persist during many years, that men who until then were religious, of chaste manners, and honest, are seen to present contrasts the most opposite. Of these abnormal contrarieties, that which has excited the most attention is the mania of robbery, which may be attributed to a disposition of mind very common among general paralytics, and in consequence of which they believe themselves rich, powerful, and masters of all they see. We have cited several examples; let us recall them briefly:—

The Baron de V——, a superior officer in an important administration, where he fulfilled well his functions, exhibited, six years before his entry into my establishment, signs of a notable alteration of his habits of life. Habitually generous, and living in excellent relations with his wife, he manifested sordid avarice, unbridled licentiousness, and ended by pilfering from his friends.

The individual who pilfered at sales, and whose conduct made a great noise at the time, was arrested, and committed to prison. When examined, his defence, which consisted in saying that he had but used a right of his profession, his placidity and carelessness, the slight impression made upon him by the judicial proceedings and the consequences of his acts, and his antecedents, until then irreproachable, led the magistrates to conclude that in his case there was mental derangement. He was compelled to resign his post, and he was confided to the care of his family; but nearly seven years passed before insanity was actually recognised.

The railway clerk who had been guilty of very considerable misuse of funds, when questioned on the subject, no suspicion having been previously entertained that his mind was diseased, said:—"I have only taken what belonged to me; this money was mine; I have gained it by my work and the improvements I

have introduced into the establishment." The reasons by which this opinion was supported, left no doubt on the already advanced derangement of his mind.

The ambitious mania is not the least curious of the phases of general paralysis. It is all the more useful to note this phase, since when present it often happens that the faculties appear to be in their natural state, no one suspecting the perturbation which exists. In the beginning, the exaggeration of self that I have particularly directed attention to, the ambitious mania, does not present itself with those well-defined and grotesque characters, which it possesses subsequently. General paralytics in embryo exhibit an exuberance of contentment and power; they speak, some of places, of dignities, and of honourable distinctions; others of speculations, of transactions, and of purchases to be effected which offer great chances of fortune. These converse of ameliorations, consummations, and discoveries, those of comedies, romances, and books to be published, the merit of which will secure their recompense. These discourses are looked upon as simple manifestations of the desires which never cease to agitate man—no singular phrases, no unaccustomed actions rousing the attention to a more serious consideration of the matter. Or if it should so happen that the personages in question become more lively, more gay, or more enterprising than usual, and deviate somewhat from their ordinary course of life, they are said to be *eccentric*, and there is an end of the matter.

An attempt has been made to circumscribe singularly this insanity of riches, this mania of grandeur, this pride of self, which Bayle had asserted to be one of the characteristic signs of paralysis of the insane, and which reveals too clearly, in its pathological expression, one of the moral wounds of the age. We have sought information upon the exactitude of Bayle's statement, and have already expressed ourselves in these terms.* "On investigating the frequency of ambitious delirium in the forty-two observations contained in the work of M. Calmeil, we have noted twenty-five which offer this type; in the eighty-five observations of Bayle, fifty-two present the symptom of mania of riches or of honours. It may have existed in other cases, because the patients were admitted only when in the third degree of the disease or upon the point of expiring. Let us observe that this delirium ought to be studied during the whole progress of the disease; because some paralytics who at the commencement of the malady have no ideas of grandeur, manifest them subsequently; and so reciprocally. Sometimes these ideas are fugacious, and show themselves as a species of lightning, so to speak. M. Baillarger

* Article, DEMENCE PARALYTIQUE, dans *Bibliothèque du Médecin Practicien* (*Maladies du Cerveau, &c.*), t. ix. p. 548.

has made the same remark. In his lectures of 1844* he says: 'It is necessary to watch the individuals during the whole course of the malady; because some persons who at the beginning of the disease have no grandiose ideas, manifest them at a later date, and *vice versâ.*' " At the sitting of the Academy of Sciences on the 17th September, he stated that he regarded this symptom as one of the principal ones of general paralysis.

"The coincidence of mania of grandeur and of general paralysis," states M. de Crozant, in his report of the patients admitted under the care of M. Voisin in the year 1841, "is a truth which I cannot understand how it has been contested. I certify that there will not be found in Bicêtre a single paralytic (I have at this moment before me the observations made upon these patients) who is not suffering from ambitious mania, provided always that the individual be fittingly examined, and that such replies as that he is neither rich nor powerful be not received as decisive.

"I conducted one day over Bicêtre a physician who applied himself to the study of mental alienation, and who held a contrary opinion, and I desired him to point out to me a solitary paralytic who did not manifest this form of mania. After much research he brought to me a patient, and, with an air of triumph, he asked him before me if he were rich, and a prince; if he were worthy of becoming one, and if he did not hope to become one. The patient responded very properly that he was a tailor, that he barely earned what sufficed for himself and family, and that he had no other desire than that of doing well his duty in his station of life. His entire conversation was very rational, and so far as it went this patient appeared to be an exception. I did not, however, delay to prove to my *confrère* that all this passed for nothing. In fact, when the patient was questioned upon his station in life, and of the manner in which he fulfilled it, we quickly learned that we had to do with the most able, the most distinguished, the most eminent tailor that ever existed—a tailor the most childish and the most superb that could possibly be interrogated. My adversary avowed that he was wrong in this instance. Paralytics carry this self-sufficiency, this presumption, into all that relates to them; their beauty, their health, their muscular force, and their talents are successively vaunted and trumpeted forth among themselves in the most pompous style—a style laden with images and epithets."†

To revert now to our peculiar consideration, the opinion of our *confrère* and friend Bayle. We have analysed one hundred observations which we have collected for the purpose of throwing right upon many controverted points of general paralysis. This is the result of our researches on the point in question:—

* *Gazette des Hôpiteaux*, 14th July. † *Ibid.* 26th April, 1842.

| | |
|---|---|
| EXPANSIVE FORM.—*First Variety.*—Mania of riches and of grandeur, predominance and persistence of these ideas. | 20 |
| *Second Variety.*—Exaggeration of self-contentment and satisfaction with everything, ideas of riches and grandeur occurring from time to time. | 22 |
| *Third Variety.*—Mania of grandeur, or of riches at long intervals, often even as flashes merely. | 10 |
| *Fourth Variety.*—Double form, expansive and oppressive, with ideas of riches and grandeur. | 12 |
| | 64 |

The symptom noted by Bayle and generalized by Crozant, (without corroborating the opinion of the latter of these practitioners, is not then a frequent phenomenon, since it is noted sixty-four times in our one hundred observations; and it may be conceived that, under the influence of this idea, paralytics would carry off things which did not belong to them.

These thoughts of riches, of power, of talent, of capacity for everything, have often the most deplorable consequences for general paralytics. This morbid state, and the intellectual debility which is the consequence of it, leaves these unfortunates at the mercy of designing men, who lead their dupes into disastrous transactions, to the great injury of the victims and their families.

Several years ago, I was consulted about a rich man in whom I quickly recognised the intellectual dispositions spoken of, although he dissimulated very well his infirmity. He was one of those patients who impose upon those around them by their apparent rationality, their specious arguments, and the observation of the habitual proprieties of life. The substratum of vanity which I readily discovered, in spite of his rational address, induced me to intimate the peril he was in to his family. I could not advise them to place the patient in an asylum, indeed they would not have followed such advice, he as yet speaking so well; but, in presence of his physician, a well-informed man, I advised the family to keep upon their guard relative to his fortune. "Take precautions," I said, "against sharpers; they would easily victimise him. The danger is great on his side, and I warn you of it, because I have seen more than one example of this kind." A year passed before I heard anything more of this gentleman. Then, one day, he was brought to me, after a scene of violence which had placed one of his relatives in danger. My prediction was verified; it was necessary to make good a deficiency of 200,000 francs.

The merchant whose case is related in the sixth observation had also in the same manner squandered 600,000 francs, and reduced his wife and children to poverty. At the termination of the sitting of the Academy of Sciences, where I had communicated this paper, one of my friends said to me, "If these facts had been known, my son-in-law would not have lost 800,000 francs, ruined my daughter, and left five children to my charge!"

It is impossible for the thoughtful physician not to remark that the mania for gold which has descended among the lowest classes of society, whilst previously it was restrained to patricians and freed-men, to the higher classes and their attendants, has become incarnated in a special madness which gives to our asylums one-fifth, one-fourth, sometimes even one-third of their inhabitants. The number of those struck by it is to be counted by thousands, and is constantly increasing. It has been pretended, as in other facts of a similar character, that this augmentation depends upon a better knowledge of the malady. We shall make but one observation upon this objection, namely—that thirty years ago there were in our private asylums more demented patients and fewer deaths, while now dementia has yielded the precedence to general paralysis, and at the same time the mortality has increased. For the rest, among the authors who have quoted facts in support of the predominance of this malady, we ought not to forget M. Moreau de Tours. Comparing the reports of Bicêtre, Charenton, and the private establishment of Esquirol, the *résumé* of those three asylums may be summed up thus:—

*First asylum*, considerable increase of the number of general paralytics for the inferior classes; *second asylum*, less sensible increase for the middle classes; *third and last asylum*, stationary condition for the superior classes.* Strange contrast! this gold, after which the most of these unfortunates have run without obtaining it, they now possess at the end in a dream! They have an abundance of it; they dispose of millions upon millions, their treasury is inexhaustible; under their happy hands everything is changed into gold and precious stones. They are princes, kings, emperors, gods! I remember a poor grocer whose business transactions were bounded to the sale of sugar, pepper, and cinnamon; he believed that he associated with all the intelligences of the age. Laced with a species of Swiss banderolle, he styled himself governor-general of the universe; distributed on all hands orders upon the Bank of France; and directed that several hundreds of millions of his works should be printed. The Emperor of China, he asserted, had read his books, and commanded that a translation should be made of them!

As if it were in bitter mockery of their lot, these Crœsuses, these

---

* *Gazette Médicale*, 4th trimestre, 1850.

princes, these geniuses, these all-powerful men, are stricken with debility so great, that even when in the prime of life, a child can push them over. Their speech is interrupted momentarily by a stutter which prevents them completing a sentence; their feeble hands tremble as they take hold of an object; their legs can barely sustain them, and in the end fail altogether; and the last stage of their malady is a brutishness unknown among animals.

The forerunners of general paralysis do not develope alone the disposition to pilfer, they also produce changes of conduct which cause the greatest astonishment among those who witness them. To the examples already related, we may add the following:—A magistrate of irreproachable manners up to the age of fifty-two years, presented at this epoch a change in his character and habits which surprised his wife and friends. From being cheerful, affectionate, and reserved, he became moody, churlish, and communicative. It was noticed that he drank, and that his reason was at times deranged. Attention was also aroused by the fact that he frequently left the house, and sought to hide whither he was going. He was watched, and it was discovered, in spite of his precautions, that he frequented houses of ill-fame. Conduct so opposed to his principles and entire life, excited the supposition that his mind was disordered, a notion which, until then, had not occurred to any one. A specialist physician was invited to dinner, the family fearing to wound the feelings of the patient by a direct visit. The play of the physiognomy was still preserved, but during the repast the physician noted occasional forgetfulness, signs of inattention, certain ambitious conceptions, a slight hesitation, and inequality of the pupils. His opinion was soon formed, and from some movements of impatience which escaped the patient, he cautioned the friends to be on their guard, as it was presumable that paralysis was in progress. A few days afterwards, great excitement rendered it necessary that the patient should be placed in an asylum.

It is evident, then, that insanity, and particularly general paralysis, can change the character of individuals, and give rise to eccentric, bad, and reprehensible acts, in opposition to their known habits. But at this point a difficulty occurs: how shall we distinguish whether these acts arise from perversity of the passions, or from disease? It has been unhappily shown by experience, that men who have acquired a reputation without stain, may, under the influence of violent emotion, give the lie to their antecedents, and commit an evil action (it is requisite to mention this, although we shall not dwell upon the subject); but it very often happens that these sudden and unforeseen lapses are the result of a mental disease. Now, in cases of this kind there are frequently precursory symptoms, *avant-couriers*, as a celebrated

English alienist physician, Dr. Forbes Winslow, has well said, in his remarkable work *On Obscure Diseases of the Brain and Disorders of the Mind.* It is these precursory symptoms which it is necessary to seek for and place in evidence. In very many cases an examination readily lays bare the mischief, and the practised physician promptly demonstrates facts which the affection of the family had misunderstood, palliated, and sought to explain. Nothing is more common, indeed, in such circumstances, than to hear relatives exclaim, "We had not noticed these things; we did not attach any importance to them; or we looked upon these actions as eccentricities, the consequence of trouble. The idea of insanity never entered our thoughts." Others will say: "Now that we think of the matter, these particulars to which you have called our attention have existed a long time." Some will add, "We mourned over his conduct, we could not comprehend it, we attributed it to the time of life, to sickness," &c.

But there are cases which take us unawares, and for which we are in nowise prepared. Under these circumstances it is requisite that the physician should be doubly careful in his researches. The index which ought to guide him in the first place is the idea of disease. In the majority of these cases, in fact, where these transformations of character, disposition, and conduct are noted, it is right to suspect general paralysis; if the age be from thirty-five to forty-five years, if there be sensual and intellectual excesses, and hereditary transmission is probable, the presumption acquires more force. But there are much more certain marks, those, to wit, furnished by disorders of the three most important functions—intelligence, motility, and sensibility. General paralysis may manifest itself by lesion of motion; it may show itself first by changes both of the intelligence and the motility combined; or it may be heralded by derangement of the understanding alone.

Before examining the characteristic symptoms of these diverse lesions, it is necessary not to lose sight of an initial phenomenon, which, although it does not possess the character of universality which Bayle attributed to it, is frequently observed, and merits consideration: I allude to congestion. In our one hundred observations it has been noted sixty times. This casualty may consist in simple giddiness, or in vertigo; it may even pass unnoticed, but most commonly it is recognised, and it is followed by grave results of a special nature. The congestion determines an enfeeblement of the intellectual powers, and occasions forgetfulness, loss of memory, and indecisions. The attention, the comparison, and the judgment lose their clearness, their precision, and their ordinary stability. If the individual be required to give a *résumé* of a transaction or to express in writing his opinion upon a disputed subject which demands thought, marked differences will be observed between the work in hand and the ordinary efforts of the person of a similar character; sometimes the writing itself will be altered. When the disease has made progress, omissions will be observed, as well as the forgetfulness of words or letters. Kindliness is often more marked than usual, and the patient enters into conversation with a confidence which at a later date will become ambitious mania. At other times, on the contrary, but more rarely, a state of sadness is observed, a tendency to melancholy or hypochondriacism. However little tact the examiner may possess, the patient expresses no astonishment that a stranger should mix himself up in his affairs.

The disorders of the muscular system are the touchstone of the disease, and often even they suffice alone to reveal the existence of general paralysis. One symptom especially may be considered as the starting-point of the series of degenerations that the patient passes through. This symptom, characteristic to the specialist physician, and which escaped but a little while back the attention of able observers, manifests itself by a passing trembling of the lip, and a slight and hardly perceptible embarrassment of the tongue, a hesitation upon one letter or word, which recurs only at long intervals. No doubt this symptom alone would not be sufficient for the diagnosis of general paralysis, but it weighs heavily in the balance; it appeals strongly to the attention, and when it is joined with other well-known symptoms, there can be no doubt. This muscular enfeeblement, in truth, extends itself over the whole system: the figure has no longer any expression, because the muscles which respond to the workings of the mind contract with difficulty, or not at all; the gait loses its ordinary erectness, and the movements lose their precision, and the pupils become unequal. A certain number of these patients recognise that they are no longer apt for the sexual functions; others, on the contrary, abandon themselves to excesses of every kind. In order to ascertain if the diminution of motility be widely distributed, require the patient to grasp the hand, and it will be felt that in many the pressure is not in relation with their apparent strength.

The sensibility may present an appreciable change. M. de Crozant has remarked this fact as existing at the beginning of general paralysis. It has been doubted, but we have confirmed it in a case of commencing paralysis seen in consultation with MM. Ferrus, Baillarger, Brochin and Carrière. Anæsthesia has been met with many times in insanity on careful examination. It is common in the second and third stage of the disease. For a long time we have experimented on our paralytics, and in nearly every case we have noted diminution of cutaneous sensibility; in some instances, indeed, there was complete loss. It is necessary also to ascertain the composition of the urine, and, by the aid of electricity, the contractility of the muscular system. Finally, in several cases we have seen amaurosis, transitory deafness, and paralysis of the sixth pair of nerves, precede several years the development of general paralysis, of which a diagnosis had been made from this epoch.

In summing up the cases recorded in this article, and the remarks to which they have led, we think that we are justified in concluding:—

1. That when individuals, at a period of life already advanced, exhibit a change of character and of conduct, and commit actions which are in complete disaccord with their principles and antecedents, we ought to surmise a perversion of their intellectual faculties.

2. This surmise becomes a certainty when there is discovered among the majority of them several of the characteristic symptoms which we have enumerated.

3. The doubt which might arise in a less-marked stage of the malady, is dissipated by prolonged observation, since ninety-five per cent. of the cases of general paralysis tend to a continual progression, and the disorder terminates by death in about the same proportion of instances.

4. Lastly, the symptoms described possess a substantial importance, because they place us upon the traces of general paralysis before the disease is yet fully declared.

---

